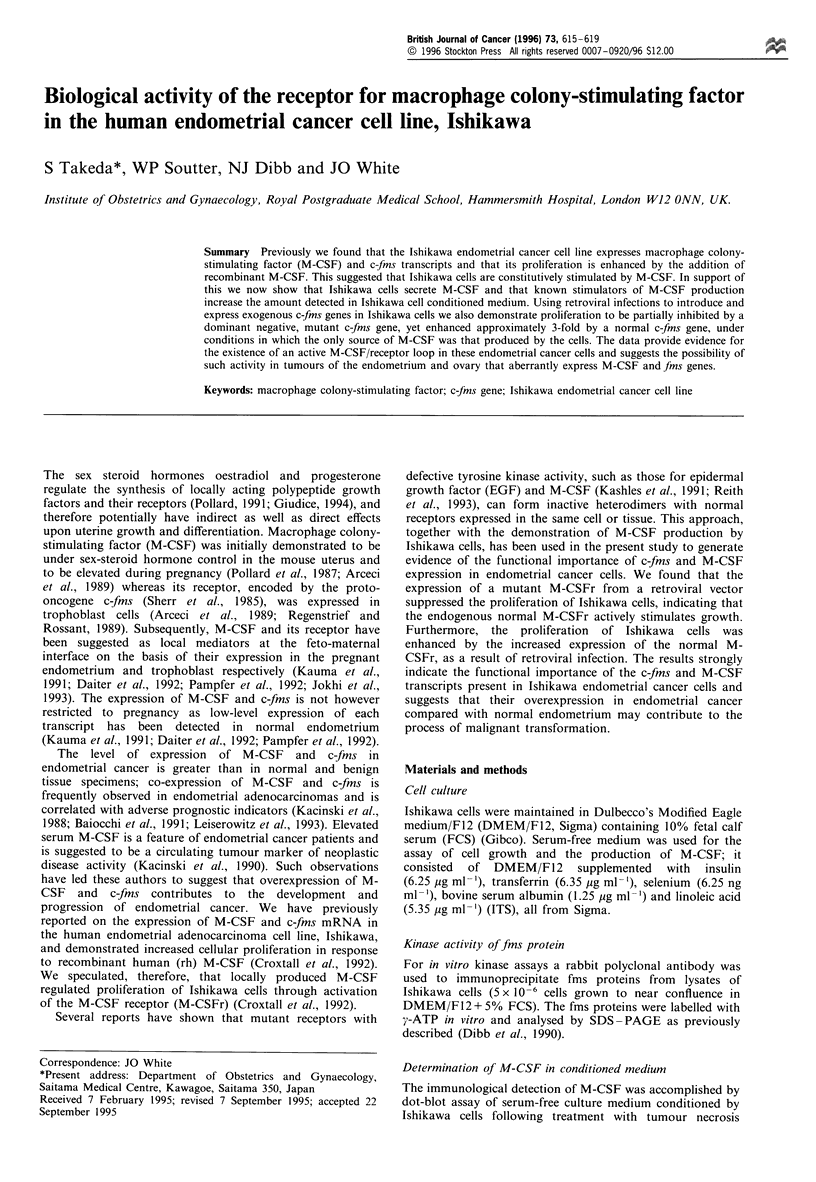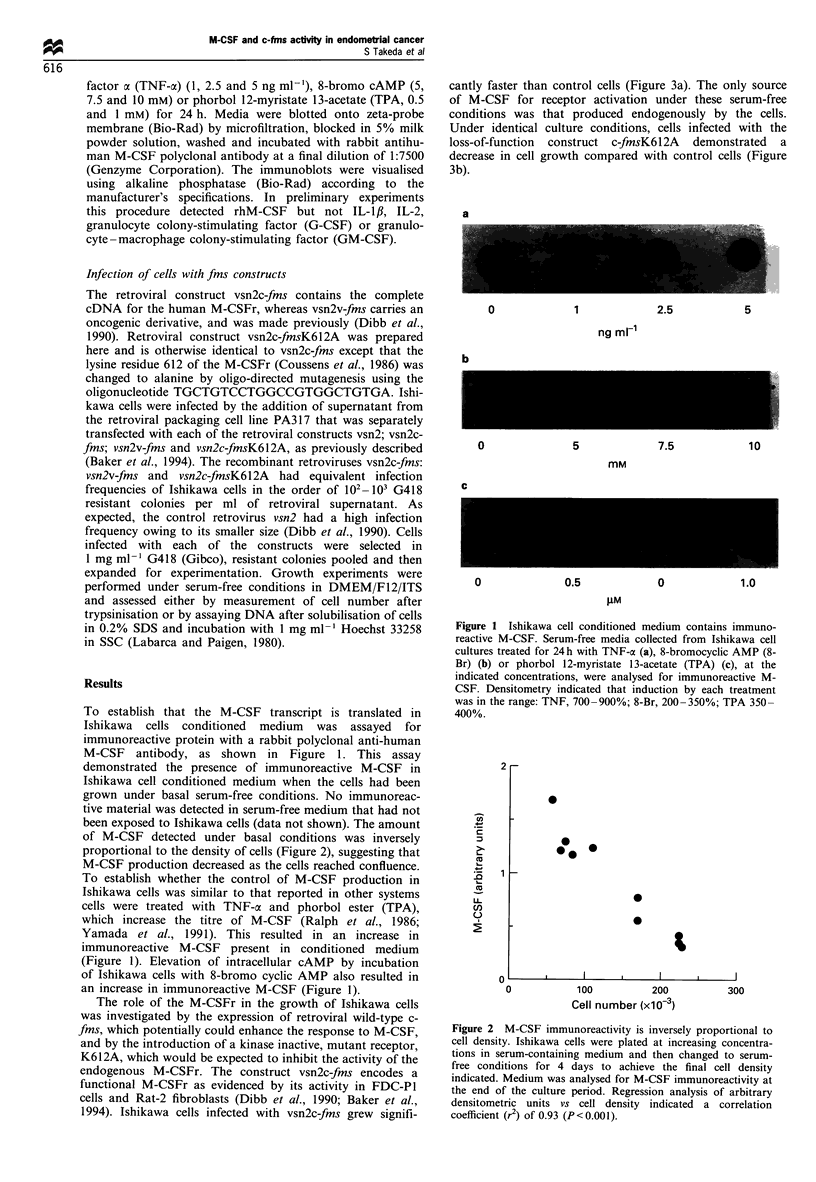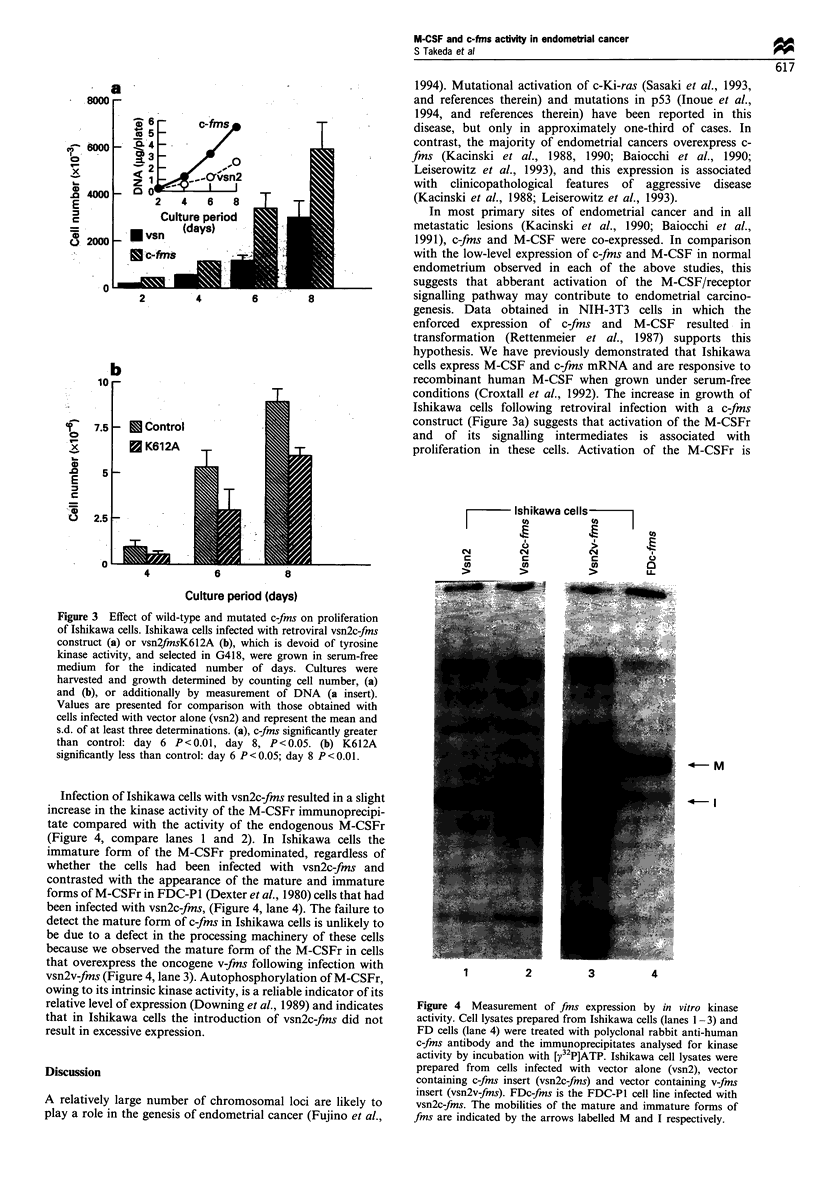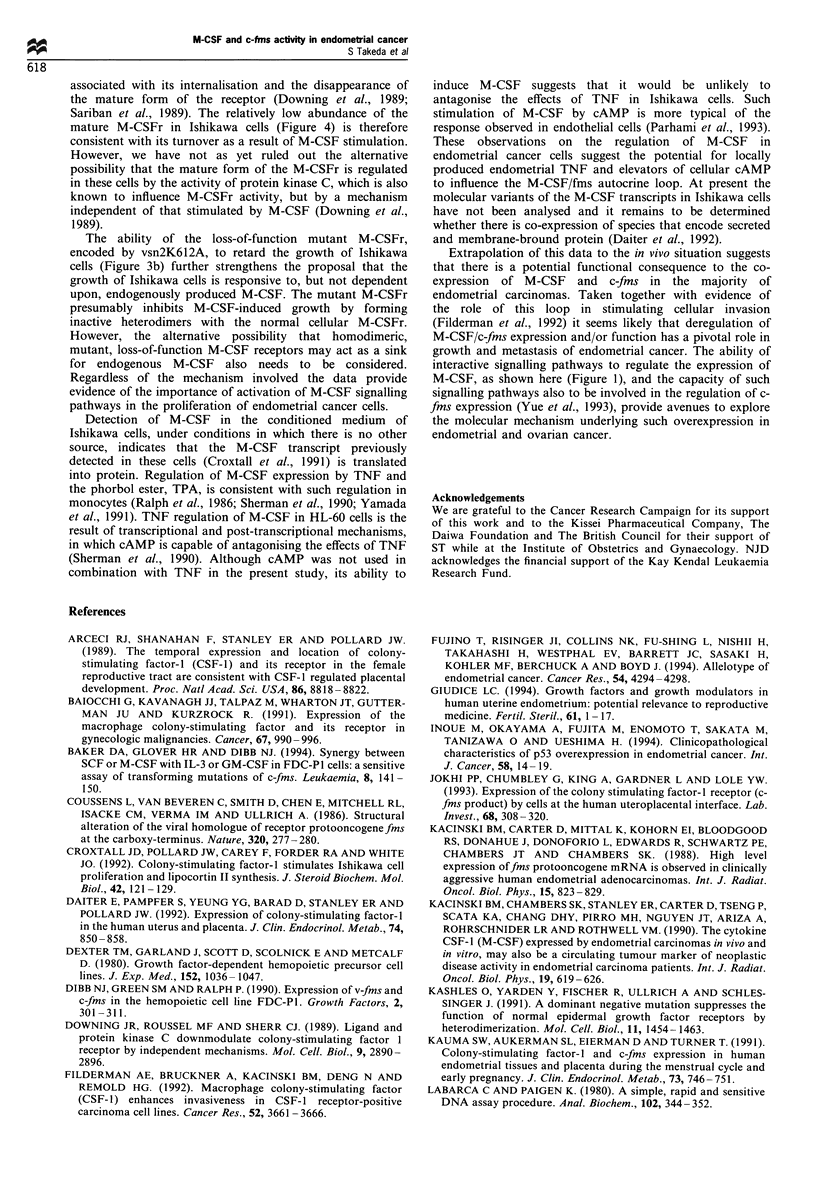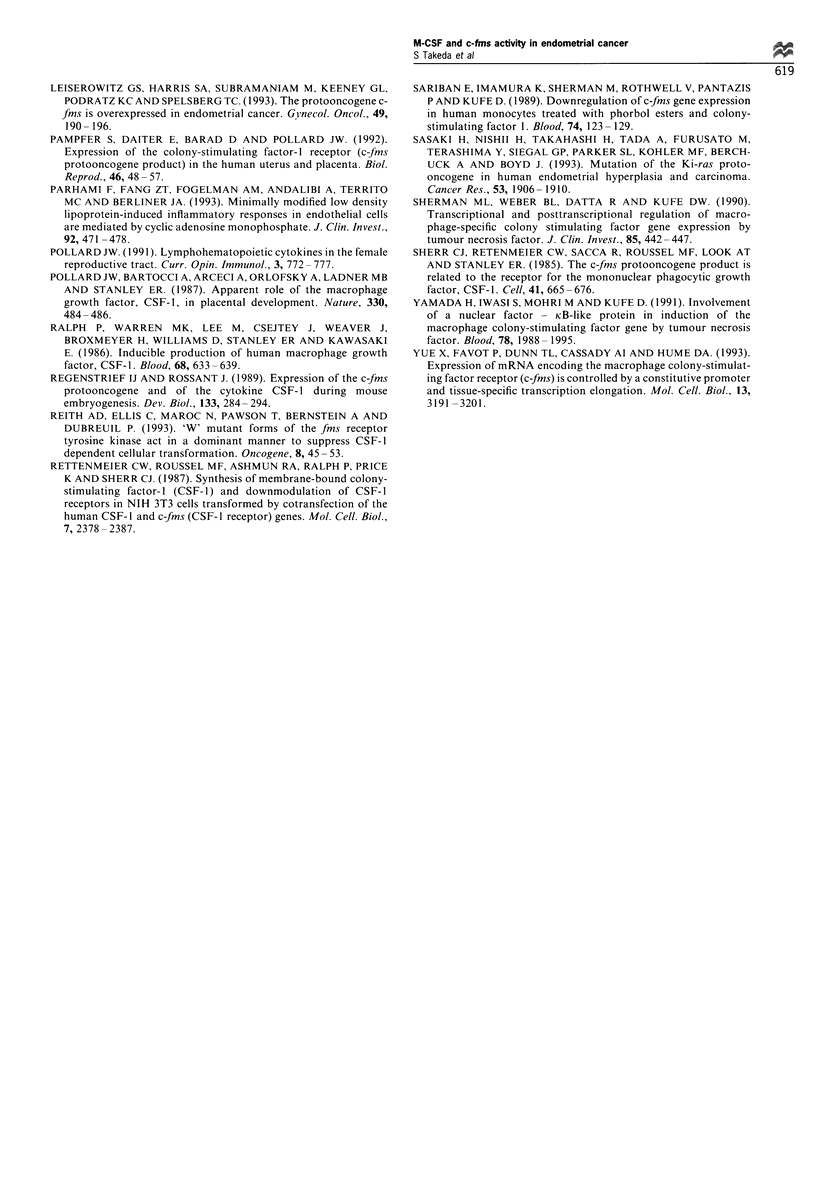# Biological activity of the receptor for macrophage colony-stimulating factor in the human endometrial cancer cell line, Ishikawa.

**DOI:** 10.1038/bjc.1996.106

**Published:** 1996-03

**Authors:** S. Takeda, W. P. Soutter, N. J. Dibb, J. O. White

**Affiliations:** Institute of Obstetrics and Gynaecology, Royal Postgraduate Medical School, Hammersmith Hospital, London, UK.

## Abstract

**Images:**


British Journal of Cancer (1996) 73, 615-619
© 1996 Stockton Press All rights reserved 0007-0920/96 $12.00

### Biological activity of the receptor for macrophage colony-stimulating factor in the human endometrial cancer cell line, Ishikawa

S Takeda*, WP Soutter, NJ Dibb and JO White

*Institute of Obstetrics and Gynaecology, Royal Postgraduate Medical School, Hammersmith Hospital, London W12 0NN, UK.*

**Summary** Previously we found that the Ishikawa endometrial cancer cell line expresses macrophage colony-stimulating factor (M-CSF) and c-*fms* transcripts and that its proliferation is enhanced by the addition of recombinant M-CSF. This suggested that Ishikawa cells are constitutively stimulated by M-CSF. In support of this we now show that Ishikawa cells secrete M-CSF and that known stimulators of M-CSF production increase the amount detected in Ishikawa cell conditioned medium. Using retroviral infections to introduce and express exogenous c-*fms* genes in Ishikawa cells we also demonstrate proliferation to be partially inhibited by a dominant negative, mutant c-*fms* gene, yet enhanced approximately 3-fold by a normal c-*fms* gene, under conditions in which the only source of M-CSF was that produced by the cells. The data provide evidence for the existence of an active M-CSF/receptor loop in these endometrial cancer cells and suggests the possibility of such activity in tumours of the endometrium and ovary that aberrantly express M-CSF and *fms* genes.

**Keywords:** macrophage colony-stimulating factor; c-*fms* gene; Ishikawa endometrial cancer cell line

The sex steroid hormones oestradiol and progesterone regulate the synthesis of locally acting polypeptide growth factors and their receptors (Pollard, 1991; Giudice, 1994), and therefore potentially have indirect as well as direct effects upon uterine growth and differentiation. Macrophage colony-stimulating factor (M-CSF) was initially demonstrated to be under sex-steroid hormone control in the mouse uterus and to be elevated during pregnancy (Pollard *et al.*, 1987; Arceci *et al.*, 1989) whereas its receptor, encoded by the proto-oncogene c-*fms* (Sherr *et al.*, 1985), was expressed in trophoblast cells (Arceci *et al.*, 1989; Regenstrief and Rossant, 1989). Subsequently, M-CSF and its receptor have been suggested as local mediators at the feto-maternal interface on the basis of their expression in the pregnant endometrium and trophoblast respectively (Kauma *et al.*, 1991; Daiter *et al.*, 1992; Pampfer *et al.*, 1992; Jokhi *et al.*, 1993). The expression of M-CSF and c-*fms* is not however restricted to pregnancy as low-level expression of each transcript has been detected in normal endometrium (Kauma *et al.*, 1991; Daiter *et al.*, 1992; Pampfer *et al.*, 1992).

The level of expression of M-CSF and c-*fms* in endometrial cancer is greater than in normal and benign tissue specimens; co-expression of M-CSF and c-*fms* is frequently observed in endometrial adenocarcinomas and is correlated with adverse prognostic indicators (Kacinski *et al.*, 1988; Baiocchi *et al.*, 1991; Leiserowitz *et al.*, 1993). Elevated serum M-CSF is a feature of endometrial cancer patients and is suggested to be a circulating tumour marker of neoplastic disease activity (Kacinski *et al.*, 1990). Such observations have led these authors to suggest that overexpression of M-CSF and c-*fms* contributes to the development and progression of endometrial cancer. We have previously reported on the expression of M-CSF and c-*fms* mRNA in the human endometrial adenocarcinoma cell line, Ishikawa, and demonstrated increased cellular proliferation in response to recombinant human (rh) M-CSF (Croxtall *et al.*, 1992). We speculated, therefore, that locally produced M-CSF regulated proliferation of Ishikawa cells through activation of the M-CSF receptor (M-CSFr) (Croxtall *et al.*, 1992).

Several reports have shown that mutant receptors with defective tyrosine kinase activity, such as those for epidermal growth factor (EGF) and M-CSF (Kashles *et al.*, 1991; Reith *et al.*, 1993), can form inactive heterodimers with normal receptors expressed in the same cell or tissue. This approach, together with the demonstration of M-CSF production by Ishikawa cells, has been used in the present study to generate evidence of the functional importance of c-*fms* and M-CSF expression in endometrial cancer cells. We found that the expression of a mutant M-CSFr from a retroviral vector suppressed the proliferation of Ishikawa cells, indicating that the endogenous normal M-CSFr actively stimulates growth. Furthermore, the proliferation of Ishikawa cells was enhanced by the increased expression of the normal M-CSFr, as a result of retroviral infection. The results strongly indicate the functional importance of the c-*fms* and M-CSF transcripts present in Ishikawa endometrial cancer cells and suggests that their overexpression in endometrial cancer compared with normal endometrium may contribute to the process of malignant transformation.

### Materials and methods

### *Cell culture*

Ishikawa cells were maintained in Dulbecco's Modified Eagle medium/F12 (DMEM/F12, Sigma) containing 10% fetal calf serum (FCS) (Gibco). Serum-free medium was used for the assay of cell growth and the production of M-CSF; it consisted of DMEM/F12 supplemented with insulin (6.25 $\mu g\ ml^{-1}$), transferrin (6.35 $\mu g\ ml^{-1}$), selenium (6.25 ng $ml^{-1}$), bovine serum albumin (1.25 $\mu g\ ml^{-1}$) and linoleic acid (5.35 $\mu g\ ml^{-1}$) (ITS), all from Sigma.

### *Kinase activity of fms protein*

For *in vitro* kinase assays a rabbit polyclonal antibody was used to immunoprecipitate fms proteins from lysates of Ishikawa cells ($5 \times 10^{-6}$ cells grown to near confluence in DMEM/F12+5% FCS). The fms proteins were labelled with $\gamma$-ATP *in vitro* and analysed by SDS-PAGE as previously described (Dibb *et al.*, 1990).

### *Determination of M-CSF in conditioned medium*

The immunological detection of M-CSF was accomplished by dot-blot assay of serum-free culture medium conditioned by Ishikawa cells following treatment with tumour necrosis

Correspondence: JO White
*Present address: Department of Obstetrics and Gynaecology, Saitama Medical Centre, Kawagoe, Saitama 350, Japan
Received 7 February 1995; revised 7 September 1995; accepted 22 September 1995

factor α (TNF-α) (1, 2.5 and 5 ng $ml^{-1}$), 8-bromo cAMP (5, 7.5 and 10 mM) or phorbol 12-myristate 13-acetate (TPA, 0.5 and 1 mM) for 24 h. Media were blotted onto zeta-probe membrane (Bio-Rad) by microfiltration, blocked in 5% milk powder solution, washed and incubated with rabbit antihuman M-CSF polyclonal antibody at a final dilution of 1:7500 (Genzyme Corporation). The immunoblots were visualised using alkaline phosphatase (Bio-Rad) according to the manufacturer's specifications. In preliminary experiments this procedure detected rhM-CSF but not IL-1β, IL-2, granulocyte colony-stimulating factor (G-CSF) or granulocyte–macrophage colony-stimulating factor (GM-CSF).

*Infection of cells with fms constructs*

The retroviral construct vsn2c-*fms* contains the complete cDNA for the human M-CSFr, whereas vsn2v-*fms* carries an oncogenic derivative, and was made previously (Dibb *et al*., 1990). Retroviral construct vsn2c-*fms*K612A was prepared here and is otherwise identical to vsn2c-*fms* except that the lysine residue 612 of the M-CSFr (Coussens *et al*., 1986) was changed to alanine by oligo-directed mutagenesis using the oligonucleotide TGCTGTCCTGGCCGTGGCTGTGA. Ishikawa cells were infected by the addition of supernatant from the retroviral packaging cell line PA317 that was separately transfected with each of the retroviral constructs vsn2; vsn2c-*fms*; *vsn2*v-*fms* and *vsn2c-fms*K612A, as previously described (Baker *et al*., 1994). The recombinant retroviruses vsn2c-*fms*: *vsn2*v-*fms* and *vsn2c-fms*K612A had equivalent infection frequencies of Ishikawa cells in the order of $10^2-10^3$ G418 resistant colonies per ml of retroviral supernatant. As expected, the control retrovirus *vsn2* had a high infection frequency owing to its smaller size (Dibb *et al*., 1990). Cells infected with each of the constructs were selected in 1 mg $ml^{-1}$ G418 (Gibco), resistant colonies pooled and then expanded for experimentation. Growth experiments were performed under serum-free conditions in DMEM/F12/ITS and assessed either by measurement of cell number after trypsinisation or by assaying DNA after solubilisation of cells in 0.2% SDS and incubation with 1 mg $ml^{-1}$ Hoechst 33258 in SSC (Labarca and Paigen, 1980).

### Results

To establish that the M-CSF transcript is translated in Ishikawa cells conditioned medium was assayed for immunoreactive protein with a rabbit polyclonal anti-human M-CSF antibody, as shown in Figure 1. This assay demonstrated the presence of immunoreactive M-CSF in Ishikawa cell conditioned medium when the cells had been grown under basal serum-free conditions. No immunoreactive material was detected in serum-free medium that had not been exposed to Ishikawa cells (data not shown). The amount of M-CSF detected under basal conditions was inversely proportional to the density of cells (Figure 2), suggesting that M-CSF production decreased as the cells reached confluence. To establish whether the control of M-CSF production in Ishikawa cells was similar to that reported in other systems cells were treated with TNF-α and phorbol ester (TPA), which increase the titre of M-CSF (Ralph *et al*., 1986; Yamada *et al*., 1991). This resulted in an increase in immunoreactive M-CSF present in conditioned medium (Figure 1). Elevation of intracellular cAMP by incubation of Ishikawa cells with 8-bromo cyclic AMP also resulted in an increase in immunoreactive M-CSF (Figure 1).

The role of the M-CSFr in the growth of Ishikawa cells was investigated by the expression of retroviral wild-type c-*fms*, which potentially could enhance the response to M-CSF, and by the introduction of a kinase inactive, mutant receptor, K612A, which would be expected to inhibit the activity of the endogenous M-CSFr. The construct vsn2c-*fms* encodes a functional M-CSFr as evidenced by its activity in FDC-P1 cells and Rat-2 fibroblasts (Dibb *et al*., 1990; Baker *et al*., 1994). Ishikawa cells infected with vsn2c-*fms* grew significantly faster than control cells (Figure 3a). The only source of M-CSF for receptor activation under these serum-free conditions was that produced endogenously by the cells. Under identical culture conditions, cells infected with the loss-of-function construct c-*fms*K612A demonstrated a decrease in cell growth compared with control cells (Figure 3b).

**Figure 1** Ishikawa cell conditioned medium contains immunoreactive M-CSF. Serum-free media collected from Ishikawa cell cultures treated for 24 h with TNF-α (**a**), 8-bromocyclic AMP (8-Br) (**b**) or phorbol 12-myristate 13-acetate (TPA) (**c**), at the indicated concentrations, were analysed for immunoreactive M-CSF. Densitometry indicated that induction by each treatment was in the range: TNF, 700–900%; 8-Br, 200–350%; TPA 350–400%.

**Figure 2** M-CSF immunoreactivity is inversely proportional to cell density. Ishikawa cells were plated at increasing concentrations in serum-containing medium and then changed to serum-free conditions for 4 days to achieve the final cell density indicated. Medium was analysed for M-CSF immunoreactivity at the end of the culture period. Regression analysis of arbitrary densitometric units *vs* cell density indicated a correlation coefficient ($r^2$) of 0.93 ($P<0.001$).

**Figure 3** Effect of wild-type and mutated c-*fms* on proliferation of Ishikawa cells. Ishikawa cells infected with retroviral vsn2c-*fms* construct (**a**) or vsn2*fms*K612A (**b**), which is devoid of tyrosine kinase activity, and selected in G418, were grown in serum-free medium for the indicated number of days. Cultures were harvested and growth determined by counting cell number, (**a**) and (**b**), or additionally by measurement of DNA (**a** insert). Values are presented for comparison with those obtained with cells infected with vector alone (vsn2) and represent the mean and s.d. of at least three determinations. (**a**), c-*fms* significantly greater than control: day 6 $P<0.01$, day 8, $P<0.05$. (**b**) K612A significantly less than control: day 6 $P<0.05$; day 8 $P<0.01$.

Infection of Ishikawa cells with vsn2c-*fms* resulted in a slight increase in the kinase activity of the M-CSFr immunoprecipitate compared with the activity of the endogenous M-CSFr (Figure 4, compare lanes 1 and 2). In Ishikawa cells the immature form of the M-CSFr predominated, regardless of whether the cells had been infected with vsn2c-*fms* and contrasted with the appearance of the mature and immature forms of M-CSFr in FDC-P1 (Dexter *et al.*, 1980) cells that had been infected with vsn2c-*fms*, (Figure 4, lane 4). The failure to detect the mature form of c-*fms* in Ishikawa cells is unlikely to be due to a defect in the processing machinery of these cells because we observed the mature form of the M-CSFr in cells that overexpress the oncogene v-*fms* following infection with vsn2v-*fms* (Figure 4, lane 3). Autophosphorylation of M-CSFr, owing to its intrinsic kinase activity, is a reliable indicator of its relative level of expression (Downing *et al.*, 1989) and indicates that in Ishikawa cells the introduction of vsn2c-*fms* did not result in excessive expression.

### Discussion

A relatively large number of chromosomal loci are likely to play a role in the genesis of endometrial cancer (Fujino *et al.*, 1994). Mutational activation of c-Ki-*ras* (Sasaki *et al.*, 1993, and references therein) and mutations in p53 (Inoue *et al.*, 1994, and references therein) have been reported in this disease, but only in approximately one-third of cases. In contrast, the majority of endometrial cancers overexpress c-*fms* (Kacinski *et al.*, 1988, 1990; Baiocchi *et al.*, 1990; Leiserowitz *et al.*, 1993), and this expression is associated with clinicopathological features of aggressive disease (Kacinski *et al.*, 1988; Leiserowitz *et al.*, 1993).

In most primary sites of endometrial cancer and in all metastatic lesions (Kacinski *et al.*, 1990; Baiocchi *et al.*, 1991), c-*fms* and M-CSF were co-expressed. In comparison with the low-level expression of c-*fms* and M-CSF in normal endometrium observed in each of the above studies, this suggests that abberant activation of the M-CSF/receptor signalling pathway may contribute to endometrial carcinogenesis. Data obtained in NIH-3T3 cells in which the enforced expression of c-*fms* and M-CSF resulted in transformation (Rettenmeier *et al.*, 1987) supports this hypothesis. We have previously demonstrated that Ishikawa cells express M-CSF and c-*fms* mRNA and are responsive to recombinant human M-CSF when grown under serum-free conditions (Croxtall *et al.*, 1992). The increase in growth of Ishikawa cells following retroviral infection with a c-*fms* construct (Figure 3a) suggests that activation of the M-CSFr and of its signalling intermediates is associated with proliferation in these cells. Activation of the M-CSFr is

**Figure 4** Measurement of *fms* expression by *in vitro* kinase activity. Cell lysates prepared from Ishikawa cells (lanes 1–3) and FD cells (lane 4) were treated with polyclonal rabbit anti-human c-*fms* antibody and the immunoprecipitates analysed for kinase activity by incubation with [$\gamma^{32}$P]ATP. Ishikawa cell lysates were prepared from cells infected with vector alone (vsn2), vector containing c-*fms* insert (vsn2c-*fms*) and vector containing v-*fms* insert (vsn2v-*fms*). FDc-*fms* is the FDC-P1 cell line infected with vsn2c-*fms*. The mobilities of the mature and immature forms of *fms* are indicated by the arrows labelled M and I respectively.

associated with its internalisation and the disappearance of the mature form of the receptor (Downing *et al*., 1989; Sariban *et al*., 1989). The relatively low abundance of the mature M-CSFr in Ishikawa cells (Figure 4) is therefore consistent with its turnover as a result of M-CSF stimulation. However, we have not as yet ruled out the alternative possibility that the mature form of the M-CSFr is regulated in these cells by the activity of protein kinase C, which is also known to influence M-CSFr activity, but by a mechanism independent of that stimulated by M-CSF (Downing *et al*., 1989).

The ability of the loss-of-function mutant M-CSFr, encoded by vsn2K612A, to retard the growth of Ishikawa cells (Figure 3b) further strengthens the proposal that the growth of Ishikawa cells is responsive to, but not dependent upon, endogenously produced M-CSF. The mutant M-CSFr presumably inhibits M-CSF-induced growth by forming inactive heterodimers with the normal cellular M-CSFr. However, the alternative possibility that homodimeric, mutant, loss-of-function M-CSF receptors may act as a sink for endogenous M-CSF also needs to be considered. Regardless of the mechanism involved the data provide evidence of the importance of activation of M-CSF signalling pathways in the proliferation of endometrial cancer cells.

Detection of M-CSF in the conditioned medium of Ishikawa cells, under conditions in which there is no other source, indicates that the M-CSF transcript previously detected in these cells (Croxtall *et al*., 1991) is translated into protein. Regulation of M-CSF expression by TNF and the phorbol ester, TPA, is consistent with such regulation in monocytes (Ralph *et al*., 1986; Sherman *et al*., 1990; Yamada *et al*., 1991). TNF regulation of M-CSF in HL-60 cells is the result of transcriptional and post-transcriptional mechanisms, in which cAMP is capable of antagonising the effects of TNF (Sherman *et al*., 1990). Although cAMP was not used in combination with TNF in the present study, its ability to induce M-CSF suggests that it would be unlikely to antagonise the effects of TNF in Ishikawa cells. Such stimulation of M-CSF by cAMP is more typical of the response observed in endothelial cells (Parhami *et al*., 1993). These observations on the regulation of M-CSF in endometrial cancer cells suggest the potential for locally produced endometrial TNF and elevators of cellular cAMP to influence the M-CSF/fms autocrine loop. At present the molecular variants of the M-CSF transcripts in Ishikawa cells have not been analysed and it remains to be determined whether there is co-expression of species that encode secreted and membrane-bround protein (Daiter *et al*., 1992).

Extrapolation of this data to the *in vivo* situation suggests that there is a potential functional consequence to the co-expression of M-CSF and c-*fms* in the majority of endometrial carcinomas. Taken together with evidence of the role of this loop in stimulating cellular invasion (Filderman *et al*., 1992) it seems likely that deregulation of M-CSF/c-*fms* expression and/or function has a pivotal role in growth and metastasis of endometrial cancer. The ability of interactive signalling pathways to regulate the expression of M-CSF, as shown here (Figure 1), and the capacity of such signalling pathways also to be involved in the regulation of c-*fms* expression (Yue *et al*., 1993), provide avenues to explore the molecular mechanism underlying such overexpression in endometrial and ovarian cancer.

**Acknowledgements**
We are grateful to the Cancer Research Campaign for its support of this work and to the Kissei Pharmaceutical Company, The Daiwa Foundation and The British Council for their support of ST while at the Institute of Obstetrics and Gynaecology. NJD acknowledges the financial support of the Kay Kendal Leukaemia Research Fund.

### References

ARCECI RJ, SHANAHAN F, STANLEY ER AND POLLARD JW. (1989). The temporal expression and location of colony-stimulating factor-1 (CSF-1) and its receptor in the female reproductive tract are consistent with CSF-1 regulated placental development. *Proc. Natl Acad. Sci. USA*, **86**, 8818–8822.

BAIOCCHI G, KAVANAGH JJ, TALPAZ M, WHARTON JT, GUTTERMAN JU AND KURZROCK R. (1991). Expression of the macrophage colony-stimulating factor and its receptor in gynecologic malignancies. *Cancer*, **67**, 990–996.

BAKER DA, GLOVER HR AND DIBB NJ. (1994). Synergy between SCF or M-CSF with IL-3 or GM-CSF in FDC-P1 cells: a sensitive assay of transforming mutations of c-*fms*. *Leukaemia*, **8**, 141–150.

COUSSENS L, VAN BEVEREN C, SMITH D, CHEN E, MITCHELL RL, ISACKE CM, VERMA IM AND ULLRICH A. (1986). Structural alteration of the viral homologue of receptor protooncogene *fms* at the carboxy-terminus. *Nature*, **320**, 277–280.

CROXTALL JD, POLLARD JW, CAREY F, FORDER RA AND WHITE JO. (1992). Colony-stimulating factor-1 stimulates Ishikawa cell proliferation and lipocortin II synthesis. *J. Steroid Biochem. Mol. Biol*., **42**, 121–129.

DAITER E, PAMPFER S, YEUNG YG, BARAD D, STANLEY ER AND POLLARD JW. (1992). Expression of colony-stimulating factor-1 in the human uterus and placenta. *J. Clin. Endocrinol. Metab*., **74**, 850–858.

DEXTER TM, GARLAND J, SCOTT D, SCOLNICK E AND METCALF D. (1980). Growth factor-dependent hemopoietic precursor cell lines. *J. Exp. Med*., **152**, 1036–1047.

DIBB NJ, GREEN SM AND RALPH P. (1990). Expression of v-*fms* and c-*fms* in the hemopoietic cell line FDC-P1. *Growth Factors*, **2**, 301–311.

DOWNING JR, ROUSSEL MF AND SHERR CJ. (1989). Ligand and protein kinase C downmodulate colony-stimulating factor 1 receptor by independent mechanisms. *Mol. Cell. Biol*., **9**, 2890–2896.

FILDERMAN AE, BRUCKNER A, KACINSKI BM, DENG N AND REMOLD HG. (1992). Macrophage colony-stimulating factor (CSF-1) enhances invasiveness in CSF-1 receptor-positive carcinoma cell lines. *Cancer Res*., **52**, 3661–3666.

FUJINO T, RISINGER JI, COLLINS NK, FU-SHING L, NISHII H, TAKAHASHI H, WESTPHAL EV, BARRETT JC, SASAKI H, KOHLER MF, BERCHUCK A AND BOYD J. (1994). Allelotype of endometrial cancer. *Cancer Res*., **54**, 4294–4298.

GIUDICE LC. (1994). Growth factors and growth modulators in human uterine endometrium: potential relevance to reproductive medicine. *Fertil. Steril*., **61**, 1–17.

INOUE M, OKAYAMA A, FUJITA M, ENOMOTO T, SAKATA M, TANIZAWA O AND UESHIMA H. (1994). Clinicopathological characteristics of p53 overexpression in endometrial cancer. *Int. J. Cancer*, **58**, 14–19.

JOKHI PP, CHUMBLEY G, KING A, GARDNER L AND LOLE YW. (1993). Expression of the colony stimulating factor-1 receptor (c-*fms* product) by cells at the human uteroplacental interface. *Lab. Invest*., **68**, 308–320.

KACINSKI BM, CARTER D, MITTAL K, KOHORN EI, BLOODGOOD RS, DONAHUE J, DONOFORIO L, EDWARDS R, SCHWARTZ PE, CHAMBERS JT AND CHAMBERS SK. (1988). High level expression of *fms* protooncogene mRNA is observed in clinically aggressive human endometrial adenocarcinomas. *Int. J. Radiat. Oncol. Biol. Phys*., **15**, 823–829.

KACINSKI BM, CHAMBERS SK, STANLEY ER, CARTER D, TSENG P, SCATA KA, CHANG DHY, PIRRO MH, NGUYEN JT, ARIZA A, ROHRSCHNIDER LR AND ROTHWELL VM. (1990). The cytokine CSF-1 (M-CSF) expressed by endometrial carcinomas *in vivo* and *in vitro*, may also be a circulating tumour marker of neoplastic disease activity in endometrial carcinoma patients. *Int. J. Radiat. Oncol. Biol. Phys*., **19**, 619–626.

KASHLES O, YARDEN Y, FISCHER R, ULLRICH A AND SCHLESSINGER J. (1991). A dominant negative mutation suppresses the function of normal epidermal growth factor receptors by heterodimerization. *Mol. Cell. Biol*., **11**, 1454–1463.

KAUMA SW, AUKERMAN SL, EIERMAN D AND TURNER T. (1991). Colony-stimulating factor-1 and c-*fms* expression in human endometrial tissues and placenta during the menstrual cycle and early pregnancy. *J. Clin. Endocrinol. Metab*., **73**, 746–751.

LABARCA C AND PAIGEN K. (1980). A simple, rapid and sensitive DNA assay procedure. *Anal. Biochem*., **102**, 344–352.

LEISEROWITZ GS, HARRIS SA, SUBRAMANIAM M, KEENEY GL, PODRATZ KC AND SPELSBERG TC. (1993). The protooncogene c-*fms* is overexpressed in endometrial cancer. *Gynecol. Oncol.*, **49,** 190–196.

PAMPFER S, DAITER E, BARAD D AND POLLARD JW. (1992). Expression of the colony-stimulating factor-1 receptor (c-*fms* protooncogene product) in the human uterus and placenta. *Biol. Reprod.*, **46,** 48–57.

PARHAMI F, FANG ZT, FOGELMAN AM, ANDALIBI A, TERRITO MC AND BERLINER JA. (1993). Minimally modified low density lipoprotein-induced inflammatory responses in endothelial cells are mediated by cyclic adenosine monophosphate. *J. Clin. Invest.*, **92,** 471–478.

POLLARD JW. (1991). Lymphohematopoietic cytokines in the female reproductive tract. *Curr. Opin. Immunol.*, **3,** 772–777.

POLLARD JW, BARTOCCI A, ARCECI A, ORLOFSKY A, LADNER MB AND STANLEY ER. (1987). Apparent role of the macrophage growth factor, CSF-1, in placental development. *Nature*, **330,** 484–486.

RALPH P, WARREN MK, LEE M, CSEJTEY J, WEAVER J, BROXMEYER H, WILLIAMS D, STANLEY ER AND KAWASAKI E. (1986). Inducible production of human macrophage growth factor, CSF-1. *Blood*, **68,** 633–639.

REGENSTRIEF IJ AND ROSSANT J. (1989). Expression of the c-*fms* protooncogene and of the cytokine CSF-1 during mouse embryogenesis. *Dev. Biol.*, **133,** 284–294.

REITH AD, ELLIS C, MAROC N, PAWSON T, BERNSTEIN A AND DUBREUIL P. (1993). 'W' mutant forms of the *fms* receptor tyrosine kinase act in a dominant manner to suppress CSF-1 dependent cellular transformation. *Oncogene*, **8,** 45–53.

RETTENMEIER CW, ROUSSEL MF, ASHMUN RA, RALPH P, PRICE K AND SHERR CJ. (1987). Synthesis of membrane-bound colony-stimulating factor-1 (CSF-1) and downmodulation of CSF-1 receptors in NIH 3T3 cells transformed by cotransfection of the human CSF-1 and c-*fms* (CSF-1 receptor) genes. *Mol. Cell. Biol.*, **7,** 2378–2387.

SARIBAN E, IMAMURA K, SHERMAN M, ROTHWELL V, PANTAZIS P AND KUFE D. (1989). Downregulation of c-*fms* gene expression in human monocytes treated with phorbol esters and colony-stimulating factor 1. *Blood*, **74,** 123–129.

SASAKI H, NISHII H, TAKAHASHI H, TADA A, FURUSATO M, TERASHIMA Y, SIEGAL GP, PARKER SL, KOHLER MF, BERCHUCK A AND BOYD J. (1993). Mutation of the Ki-*ras* protooncogene in human endometrial hyperplasia and carcinoma. *Cancer Res.*, **53,** 1906–1910.

SHERMAN ML, WEBER BL, DATTA R AND KUFE DW. (1990). Transcriptional and posttranscriptional regulation of macrophage-specific colony stimulating factor gene expression by tumour necrosis factor. *J. Clin. Invest.*, **85,** 442–447.

SHERR CJ, RETENMEIER CW, SACCA R, ROUSSEL MF, LOOK AT AND STANLEY ER. (1985). The c-*fms* protooncogene product is related to the receptor for the mononuclear phagocytic growth factor, CSF-1. *Cell*, **41,** 665–676.

YAMADA H, IWASI S, MOHRI M AND KUFE D. (1991). Involvement of a nuclear factor – κB-like protein in induction of the macrophage colony-stimulating factor gene by tumour necrosis factor. *Blood*, **78,** 1988–1995.

YUE X, FAVOT P, DUNN TL, CASSADY AI AND HUME DA. (1993). Expression of mRNA encoding the macrophage colony-stimulating factor receptor (c-*fms*) is controlled by a constitutive promoter and tissue-specific transcription elongation. *Mol. Cell. Biol.*, **13,** 3191–3201.